# Draft Genome Sequence of Lactobacillus rhamnosus NCB 441, Isolated from Egyptian White Domiati Cheese

**DOI:** 10.1128/MRA.01191-20

**Published:** 2020-12-03

**Authors:** Madison A. Moore, Hunter D. Whittington, M. Andrea Azcarate-Peril, José M. Bruno-Bárcena

**Affiliations:** a Department of Plant and Microbial Biology, North Carolina State University, Raleigh, North Carolina, USA; b Department of Medicine, Division of Gastroenterology and Hepatology, School of Medicine, University of North Carolina at Chapel Hill, Chapel Hill, North Carolina, USA; c UNC Microbiome Core, Center for Gastrointestinal Biology and Disease, School of Medicine, University of North Carolina at Chapel Hill, Chapel Hill, North Carolina, USA; Portland State University

## Abstract

Here, we report the draft genome sequence of Lactobacillus rhamnosus NCB 441, which was isolated from pickled white cheese samples gathered at Farafra Oasis in New Valley Governorate, Egypt. The genome size is 2,969,245 bp with a G+C content of 46.7%.

## Abstract (precis)

Here, we report the draft genome sequence of Lactobacillus rhamnosus NCB 441, which was isolated from pickled white cheese samples gathered at Farafra Oasis in New Valley Governorate, Egypt. The genome size is 2,969,245 bp, with a G+C content of 46.7%.

## ANNOUNCEMENT

Lactic acid bacteria inhabit a wide variety of ecological niches in addition to being members of a phylogenetically diverse group of organisms (1, 2). Of those, *Lactobacillus* is one of the more diverse genera and contains multiple commercially exploited Lactobacillus rhamnosus strains. Here, we report the draft genome sequence of L. rhamnosus NCB 441, which was isolated from pickled white cheese (Egyptian white Domiati cheese) samples gathered at Farafra Oasis in New Valley Governorate, Egypt (27.0567°N, 27.9703°E), where daily ambient temperatures reach upwards of 40°C (104°F).

Five samples of Domiati cheese were used to isolate lactic acid bacteria. Twenty-five grams of each cheese sample was homogenized with 225 ml of 0.85% NaCl, plated onto MRS agar, and incubated anaerobically at 30°C for 48 h for colony selection. Isolated colonies were inoculated into MRS broth and cultivated at 30°C prior to genomic DNA extraction. Extraction was carried out according to the method described by Hoffman and Winston (3). Briefly, cells were harvested in the early logarithmic stage of growth at an optical density at 600 nm (OD_600_) of approximately 0.4 and then were homogenized with a bead beater. The DNA was subjected to chloroform extraction and ethanol precipitation prior to library preparation. The sequencing library was produced using the 454 FLX Titanium rapid library kit according to the manufacturer’s instructions (Roche, Indianapolis, IN, USA). The Microbiome Core at the University of North Carolina at Chapel Hill generated shotgun sequencing data for the strain using a 454 GS FLX Titanium+ system (Roche).

For all software, default parameters were used except where otherwise noted. Sequencing data generated 2,607,325 raw reads which were quality filtered for a minimum 500-bp read length. Reads were assembled *de novo* using Newbler v2.6 (4) to produce 108 contigs with an *N*_50_ value of 59,695 bp and a coverage depth of 36×. The 108 contigs were subjected to a second round of assembly using SPAdes v3.14.1 (5) to produce 45 contigs with an *N*_50_ value of 111,618 bp. The resulting genome assembly of L. rhamnosus NCB 441 is 2,969,245 bp, with a G+C content of 46.7%. Genome completeness was assessed using CheckM v1.0.18 (6) and was determined to be 98.91%.

**FIG 1:**
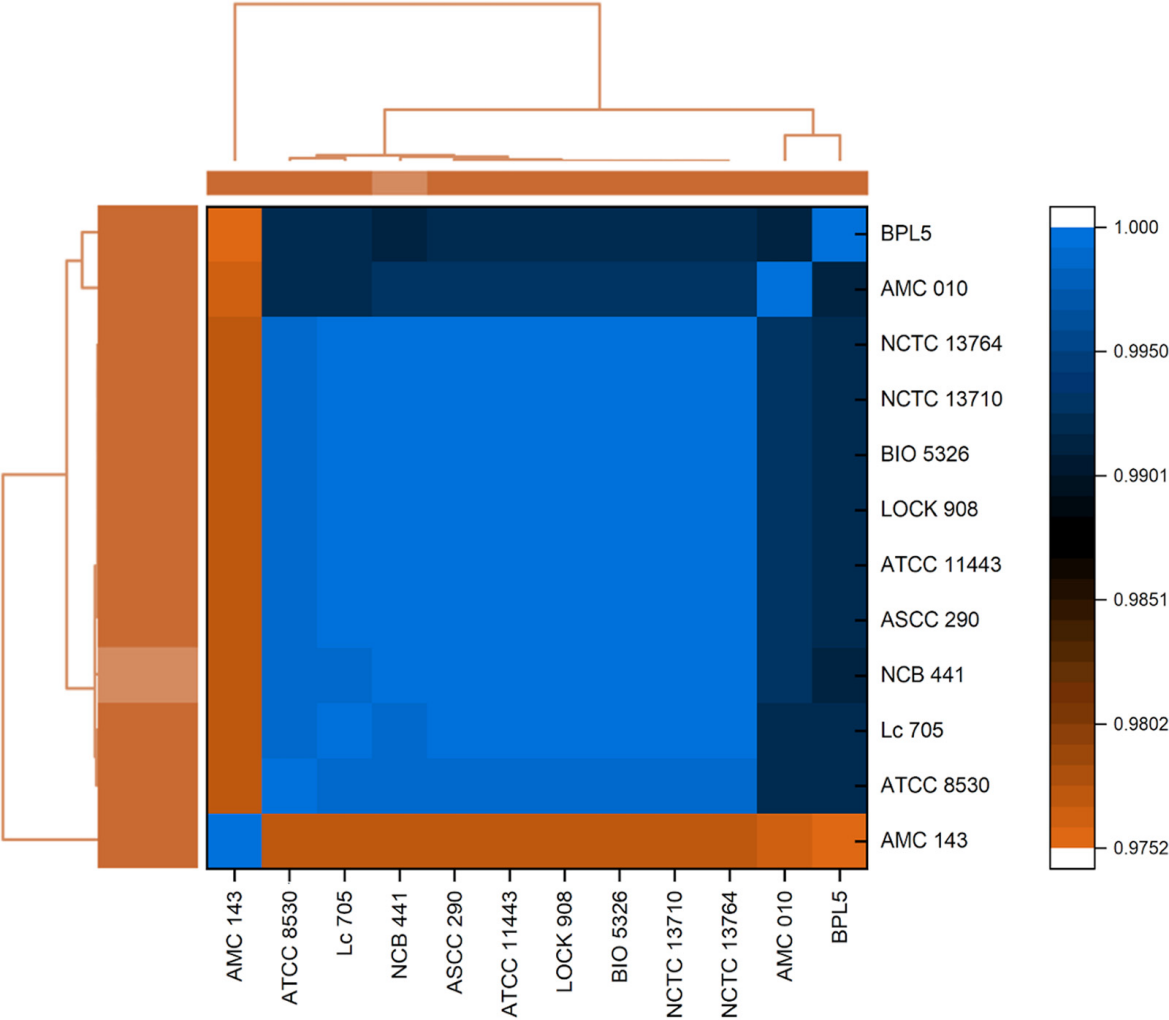
Heatmap display of ANI values for 12 Lactobacillus rhamnosus strains. The heatmap was generated with Origin v2018b using ANI values for 12 of 192 L. rhamnosus strains, including ATCC 8530 (GenBank accession no. CP003094.1), Lc 705 (FM179323.1), ASCC 290 (CP014645.1), ATCC 11443 (CP022109.1), LOCK908 (CP005485.1), BIO5326 (CP046267.1), NCTC13710 (LR134322.1), NCTC13764 (LR134331.1), BPL5 (LT220504.1), AMC010 (MSTC00000000.1), and AMC143 (MSTB00000000.1), for which sequences were obtained from NCBI. Values range from 0.9752 (97.52%) to 1 (100% ANI); orange represents <98.51% ANI, while blue represents highly similar strains exhibiting ANI values of >99%. Dendrograms link ANI percentages to construct a hierarchical clustering inferring phylogeny, thus confirming NCB 441 as a L. rhamnosus strain.

Species assignment was based on the average nucleotide identity (ANI) (7) of NCB 441 being over 97% with respect to all 192 L. rhamnosus species sequenced and deposited in the NCBI GenBank database to date. All genomes were downloaded using the command line package Pyani (8) and later were compared to NCB 441 using the MUMmer nucmer algorithm to calculate ANI values (9). A condensed heatmap was generated using Origin v2018b (OriginLab Corp., Northampton, MA) (Fig. 1) with only the top ANI values from nine fully sequenced L. rhamnosus genomes (ATCC 8530, Lc 705, ASCC 290, ATCC 11443, LOCK908, BIO5326, NCTC13710, NCTC13764, and BPL5), as well as two strains previously sequenced by our research group (AMC010 and AMC143) (10, 11). ANI values indicate over 97% nucleotide identity to strains BPL5, AMC010, and AMC143, as well as over 99% nucleotide identity to all other strains, which provides evidence for NCB 441 to be classified as a L. rhamnosus strain.

### Data availability.

The genome sequence of L. rhamnosus NCB 441 has been deposited in DDBJ/EMBL/GenBank under the accession no. JACSDP000000000. The version described in this paper is the first version. Raw sequencing data have been deposited with SRA accession no. SRR12515116.
